# An Overlooked Factor in the Production of Conjunctivitis

**Published:** 1892-07

**Authors:** Julius Pohlman

**Affiliations:** 367 Franklin Street; Buffalo, N. Y.


// Buffalo Medical and Surgical Journal

VOL. XXXI. JULY, 1892. No. 12.

### Original Communications.

### AN OVERLOOKED FACTOR IN THE PRODUCTION OF CONJUNCTIVITIS.[1]

BY JULIUS POHLMAN, M. D., Buffalo, N. Y.

WE ARE told by ophthalmologists that the eye when exposed to artificial illumination depends upon three distinct qualities of the light for its well-being, namely, (1) its whiteness, (2) its steadiness, and (3) the amount of heat which it radiates.

Hardly anybody will dare to underestimate the importance of the whiteness and the steadiness of a light. Optic nerves are organs specialized to receive sunlight, and hence the nearer the artificial illumination resembles it, *i. e.*, the whiter it is, the more the eyes remain in their natural condition; and the labor of the optic nerve, to adapt itself to the artificial surroundings, by means of muscular exertions, is reduced to a minimum to the advantage of the eye as a whole.

When we consider the function of the iris, to regulate the quantity of light which is to strike the retina and to reduce the variations of that quantity to the minimum, we can understand how a flickering flame, no matter how white, necessitates an immense strain upon this delicate muscular apparatus, a strain which increases with the unsteadiness of the light.

The third factor, however, the heat which the light radiates, in its influence upon the eye, does not seem so plain. We are taught that heat affects the conjunctiva, and that conjunctivites are often found in occupants of poorly ventilated rooms where hot lights are burning more or less all day; and that certainly seems to demonstrate the correctness of the theory. Nevertheless, how *heat*, *i. e.*, a simple difference in temperature, should injure the conjunctiva enough to produce pathological conditions, has never seemed quite

1. Read at the meeting of the Alumni Association, Buffalo University Medical College, May 3, 1892.

clear to the writer, because, if this depended merely upon a quantity of *heat*, then why should a temperature of 75° or 80°, produced by gas flames injure the conjunctiva, when a summer heat of 95 or 100°, or even more, does not? If simply a question of temperature only, then the effects ought to be the same, no matter what the source of the heat, because the whole eye is subjected equally to its influence and not a small part of it alone; were it otherwise, we may defend the apparent difference in the effects of natural and artificial heat on the same lines as we differentiate between wind and draught: one striking the whole body, the other only a circumscribed portion of it, thus having differing conditions with differing results. But this line of reasoning cannot be applied to the eye. As, however, the effect produced on the eye by the heat of artificial light *does* differ from that produced by natural heat, we must look for other or additional causes which injure the conjunctiva.

Dr. Frank P. Vandenbergh, for many years City Chemist of Buffalo, informs me that the manufacture of light gas in this city is rather behind the times; made from bituminous coal by the old-fashioned process of dry distillation, it always contains a considerable quantity of sulphur; for instance, the average of numerous analyses shows that the product of the Buffalo Gas Light Company has 12.08 grains of sulphur in every 100 cubic feet of gas; the Mutual Company, 12.93 grains, and the Citizens' Company, 9.68 grains. That means that for every 100 cubic feet of gas burned, ten to twelve grains of surphur are given to the air in the form of fumes, which, as we all know, have a special affinity for moist surfaces forming their sulphurous acid.

Several years ago the leading librarians of the country made quite extensive reports upon the corroding influence of gas light upon the leather bindings of books. If the sulphur compounds can corrode leather in the course of time in large well-ventilated libraries, are we not justified in assigning some influence to them when acting on the moist conjunctiva in a poorly-ventilated room with gas burning, perhaps, all day, or throughout the time when the eyes are exposed to the heat of the flame?

It may be quite appropriate here to mention that the natural gas used in Buffalo contains no sulphur, and will, therefore, with the right kind of burner, produce a light superior to that of artificial gas as far as any injurious effect upon the conjunctiva is concerned.

More important, however, to the eye than this small amount of sulphur is the quantity of moisture which air holds under varying temperatures. It is an old and well-known fact that the hotter the air, the larger the amount of water which it contains, and the lower the temperature the smaller the amount, all other things being equal. Supposing a room contains a certain amount of moisture at 50° when the outside air is 40°, then, if we raise the temperature of the room to 70°, we must supply a certain amount of water for evaporation in order to have the air and warmth feel agreeable, *i.e.*, not too dry; for the outside air coming in at a much lower temperature is still drier than that contained in the room originally, and we must resort to artificial means in order to supply the demand.

Artificial heat in its drying effect upon furniture, doors, etc., has often been discussed, but it seems that such discussions have always stopped at dead matter and have never been applied to living bodies.

To investigate the effects of the *dryness* of the air on the conjunctiva, the following experiments were made:

The room used was 12 x 13 feet, 9 feet 6 inches high, and heated by natural gas burned in an open front Jewett gas burning stove; this particular kind of stove has a water-tank at its back which holds two quarts of water, and forms part of the structure, so that the flames play directly against one side of it. When running at full blast, to keep the room at 65° to 70°, the contents of the tank evaporated in about six hours; in other words, it took about two gallons of water in twenty-four hours to keep the air of the room supplied with the moisture necessary for its temperature when it was freezing outside.

The ventilation of the room and the draught of the stove were perfect, and no product of combustion, complete or incomplete, or other gases of any kind from leaks in pipes or otherwise, could enter the room and mix with the air; all sources of error in this direction were avoided as carefully as possible, so that the effects produced upon the eyes of the occupants of the room were due solely to the peculiar condition of its atmosphere and to nothing else.

The experiments were made at thirty different times, lasting from thirty minutes to two hours each, upon the writer's own person. The effects produced were repeatedly corroborated by visitors who happened to call at such times, and who knew nothing about the experiments to which they were unconsciously subjected during their visits; hence, their complaints would have to be called entirely unbiased and unprejudiced, but their symptoms were always identical with those experienced by the writer.

With a temperature of 65° or 70° when the water-tank on the stove was empty and the air of the room relatively dry, an exposure of fifteen minutes made the eyes feel dry and sticky; if prolonged for another fifteen minutes, or if the temperature was raised to 80° or 85°, the symptoms became more decided,—the peculiar sensation of a foreign body in the eye, like a particle of sand or some stringy substance, became very strong. The conjunctival vessels of the lids looked engorged, the more the longer the exposure, and after a stay in a dry atmosphere of 80° for about two hours, the conjunctival vessels of the bulb were equally well marked, and the eye presented in every symptom a well-developed case of acute conjunctivitis.

While cold has a stimulating effect upon the lachrymal gland, heat apparently does not act in that way; whether it has an inhibitory function, is not yet decided, but it certainly does not increase the secretion, and though the ordinary amount of lachrymal fluid is sufficient to keep the eye moist and clean under ordinary conditions, the gland does not secrete fluid enough when the air is too dry for its temperature, and hence calls for a more rapid evaporation from all moist surfaces. The eye tries hard to overcome this deficiency by increased winking, but to no avail; evaporation from the conjunctiva proceeds quicker than the moisture is supplied by the lachrymal gland, and its surface becomes proportionally dry, producing after awhile all the disagreeable sensations of conjunctival troubles.

Whether the mechanical irritation produced by the friction of the dry conjunctival surfaces has the effect of dilating the capillaries and producing in this wise a slowing of the blood stream with the attending phenomenon of engorgement, or whether the heat has such an effect upon the partially dry eye which it would not have upon the normally moist organ, or whether there are other causes which, by themselves or in addition to those mentioned, produce the engorgement, must be left for additional investigations to decide, but that the acute conjunctivitis, mild or severe, was caused by the *dryness* of the air and not by the *heat*, was demonstrated during every experiment as follows:

Whenever, during the course of an experiment, the water-tank on the stove was filled, and free and rapid evaporation restored the equilibrium between heat and moisture, then, no matter whether the temperature of the room was 65° or 85°, the annoying symptoms disappeared in a few minutes if the exposure had not been too prolonged. In the early stages of the experiments, just when the eye commenced to feel sticky, a simple dropping of a few drops of water between the lids caused an almost instantaneous disappearance of the distressing sensations, but they returned equally promptly, and with unvarying certainty, if the eye was again exposed to the dry air. If bathed continually, no matter how hot or how dry the air, the eye felt as comfortable as under normal conditions, and none of the symptoms appeared as long as the conjunctiva was supplied with the required moisture.

What happens in the room as a whole, will take place on a smaller scale in the vicinity of a lamp,--the temperature is raised and the air is dried correspondingly, and whenever the eyes are near enough to the flame to feel its heat, they will be affected by the dryness of the air, and the hotter the light the dryer will be the air that surrounds it.

If additional observations prove this theory to be correct, then we can understand why a summer temperature of 95° or 100°, holding its normal quantity of moisture, has no effect on the eye, while artificial heat of 70°, with its usual corresponding dryness, can produce very decided forms of conjunctival troubles, and we learn that a little closer attention to the water-pan on the furnace or on the stove, is as necessary to the well-being of our eyes as the efficient lighting of the room. And if repeated experiments finally develop the fact that heat has no effect upon the conjunctiva, then we have to modify our teaching regarding the well-being of our eyes under artificial illumination, and name as the three factors necessary, whiteness and steadiness of the light, and moisture in the air surrounding the flame proportionate to its temperature.

367 Franklin Street.

---

Pie-Plant and Hematuria.—A number of cases of urinary trouble following the free eating of rhubarb, or pie-plant, have been reported. They are attended by the symptoms of oxaluria—frequent and painful micturition, hematuria, and lumbar pain. The trouble arises from the oxalic acid contained in the rhubarb uniting with the calcium of drinking water, and a consequent formation of the oxalate crystals in the uriniferous tubules.—*Doctor's Weekly.*

